# Explaining socioeconomic inequalities in immunisation coverage in India: new insights from the fourth National Family Health Survey (2015–16)

**DOI:** 10.1186/s12887-020-02196-5

**Published:** 2020-06-16

**Authors:** Swati Srivastava, Jasmine Fledderjohann, Ashish Kumar Upadhyay

**Affiliations:** 1 International Institute for Population Sciences, Mumbai, 400088 India; 2 Department of Sociology, Lancaster University, Lancaster, UK

**Keywords:** Immunisation, India, National family health survey, Concentration index, Decomposition analysis, Standardization, Immunisation intensity, Sustainable development goals

## Abstract

**Background:**

Childhood vaccinations are a vital preventive measure to reduce disease incidence and deaths among children. As a result, immunisation coverage against measles was a key indicator for monitoring the fourth Millennium Development Goal (MDG), aimed at reducing child mortality. India was among the list of countries that missed the target of this MDG. Immunisation targets continue to be included in the post-2015 Sustainable Development Goals (SDG), and are a monitoring tool for the Indian health care system. The SDGs also strongly emphasise reducing inequalities; even where immunisation coverage improves, there is a further imperative to safeguard against inequalities in immunisation outcomes. This study aims to document whether socioeconomic inequalities in immunisation coverage exist among children aged 12–59 months in India.

**Methods:**

Data for this observational study came from the fourth round of the National Family Health Survey (2015–16). We used the concentration index to assess inequalities in whether children were fully, partially or never immunised. Where children were partially immunised, we also examined immunisation intensity. Decomposition analysis was applied to examine the underlying factors associated with inequality across these categories of childhood immunisation.

**Results:**

We found that in India, only 37% of children are fully immunised, 56% are partially immunised, and 7% have never been immunised. There is a disproportionate concentration of immunised children in higher wealth quintiles, demonstrating a socioeconomic gradient in immunisation. The data also confirm this pattern of socioeconomic inequality across regions. Factors such as mother’s literacy, institutional delivery, place of residence, geographical location, and socioeconomic status explain the disparities in immunisation coverage.

**Conclusions:**

In India, there are considerable inequalities in immunisation coverage among children. It is essential to ensure an improvement in immunisation coverage and to understand underlying factors that affect poor uptake and disparities in immunisation coverage in India in order to improve child health and survival and meet the SDGs.

## Background

Over the past several decades, there has been a significant reduction in child mortality rates across the world. Globally, the under-five mortality rate has declined from 90.6 deaths per 1000 live births in 1990 to 42.5 deaths per 1000 live births in 2015 [1]. Proportionally and in absolute numbers, more childhood deaths occurred in low- and middle-income countries (LMICs) than elsewhere in the world [2, 3]. An important factor for preventing diseases and death in childhood is immunisation, which has played a critical role in the drop in child mortality rates globally. Arising from the efficacy of immunisations in improving health and reducing mortality, the United Nations set childhood immunisation coverage as a key indicator to monitor the fourth Millennium Development Goal (MDG-4), which aimed to reduce the under-five mortality rate by two-thirds between 1990 and 2015. While there was substantial progress on this goal, ultimately it was not achieved. The post-2015 Sustainable Development Goals (SDGs) thus continue to target mortality reductions through 2030. SDG 3 aims to ensure health and well-being for all, including achievement of universal immunisation coverage. The new Sustainable Development Agenda also places a strong emphasis on reducing inequalities. Monitoring not only of immunisation coverage, but also of inequalities, is therefore essential for meeting the new SDGs.

India was among the list of countries that did not meet MDG-4, and the latest evidence suggests that only 62% of children aged 12–23 months have been fully immunised—that is, that they’ve received BCG, measles, and 3 doses of both the polio and diphtheria, pertussis and tetanus (DPT) vaccines. This is far from the universal coverage targeted by SDG 3. However, improving immunisation coverage has long been a public health goal in India. The Government of India launched the Universal Immunisation Programme (UIP) in 1985 to prevent infant and child mortality from six preventable diseases: Tuberculosis, Diphtheria, Pertussis, Tetanus, Poliomyelitis and Measles [4]. The UIP specifies that by 12 months of age, children should be fully immunised. More recently, Hepatitis B was also included under the UIP in India. Details for the recommended timing for these vaccinations are presented in Table 1.

**Table 1:** Immunisation schedule according to IAP-2013

**BCG (**Bacillus Calmette–Guérin)**-** At birth or as early as possible till one-year age	
**Hepatitis B (Birth dose)-** At birth or as early as possible within 24 h	
**OPV Zero dose-** At birth or as early as possible within 15 days	
**Measles-** 9 completed months-12 months (can administer up to 5 years if not received at 9–12 months)	
**OPV** (1,2 & 3), **DPT** (1,2 & 3) and **Hepatitis B** (1,2 & 3)- At 6 weeks, 10 weeks and 14 weeks	

Meanwhile, pioneering strategies such as the Pulse Polio campaign were introduced to reduce the incidence of polio in India. As a result, India faced a drastic decline in polio incidence by the end of 2012, especially among marginalized populations [5]. It was certified as polio-free by the WHO in 2014, and has since retained that status [6]. Less progress has been made on other diseases of childhood. A substantial number of child deaths in India occur due to vaccine-preventable diseases—particularly measles, Hepatitis B and Haemophilus influenza type b (Hib) [7]. In 2011, the Hib vaccine was introduced for the first time in two southern states, in combination with DPT and Hepatitis B. Following on from this, in 2015 the Hib-conjugate vaccine was introduced under the UIP for the entire national population.

Corresponding to these government efforts, progress has been made, but full immunisation coverage is still low. The National Family Health Survey (NFHS), a repeated cross-sectional national survey and a key source of information on immunisation coverage in India, shows that full immunisation coverage has increased from 35% in the NFHS-1 (1992–93) data to 62% in the NFHS-4 (2015–16). Substantial variation was reported in coverage of specific doses of vaccines. Estimates from the NFHS-4 (2015–16) show that, for children aged 12–23 months, coverage of Hepatitis B vaccine was lowest (63%), while the highest coverage was reported for BCG (92%) vaccination. The NFHS-4 also indicates that full immunisation coverage varies considerably according to geographic region, place of residence, and socioeconomic status. Full coverage was lowest in Nagaland (35%), a state in the Northeast of India, and was highest in Puducherry (95%) in the South. Using a sub-sample (*n* = 9582) of data from the NFHS-3 (2005–06), Lauridsen and Pradhan reported socioeconomic inequalities associated with child immunisation in India [8]. Another study using the District Level Household and Facility Survey (2007–08), also documented socioeconomic disparities in coverage of full immunisation in India in a selected sub-sample (*N* = 11,212) [9]. A growing number of studies have examined socioeconomic inequalities in child immunisation in LMICs, including India. However, the pathways through which inequalities occur remain unclear. Very few studies from India have systematically examined socioeconomic inequalities in child immunisation, and previous work on this topic in India has tended to analyse selective sub-samples. Therefore, using large-scale survey data from the most recent round of the NFHS (2015–16), in this study we examine inequalities in immunisation coverage for children aged 12–59 months in India, focusing on three immunisation categories: full, partial and no immunisation coverage. We also assess socioeconomic inequalities in the intensity of immunisation coverage in India, and document some of the other key socio-demographic correlates of disparities in coverage of child immunisation.

## Methods

### Data

We used secondary survey data from the NFHS-4 (2015–16), which is the Demographic and Health Survey (DHS) for India. The principal objective of this large-scale, nationally representative household survey is to provide district, state and national level estimates on fertility, mortality, and family planning; full details of the sampling design and survey instrument are available elsewhere [10]. The 2011 census served as the sampling frame for the selection of primary sampling units (PSUs): PSUs were villages in rural areas and Census Enumeration Blocks (CEBs) in urban areas. In the first stage, villages were selected from rural areas and CEBs were selected from urban areas using a Probability Proportional to Size (PPS) sampling scheme. In every selected rural and urban PSU, a complete household mapping and listing were conducted before the survey. Selected PSUs with an estimated number of at least 300 households were divided into segments of approximately 100–150 households. Two of the segments were randomly selected for the survey using systematic PPS sampling. Therefore, an NFHS-4 cluster is either a PSU or a segment of a PSU. In the second stage, in every selected rural and urban cluster, 22 households were randomly selected with systematic sampling.

This study is based on the 182,552 children aged 12–59 months born in the 5 years preceding the survey. We excluded children below 12 months of age as they are still in the process of receiving vaccinations within the recommended time frame and thus are not yet eligible to fulfil the criteria of full immunisation.

### Dependent variables

The dependent variables of interest for our analysis are three binary indicators of immunisation status: Full immunisation (no, yes), partial immunisation (no, yes), and no immunisation (no, yes). ‘Full immunisation’ refers to children aged 12–59 months who received all 13 recommended vaccines (given in Table 1); ‘partially immunised child’ indicates children received at least one but not all recommended vaccines; and ‘non-immunised child’ indicates children did not receive any vaccines since birth.

In addition, because vaccines received by partially immunized children may range in number from 1 to 12, we also created a fourth variable, immunisation intensity, for these children. Immunisation intensity is defined as the proportion of vaccines the child has received relative to the number of vaccines the child should have received—that is, the number of vaccines received divided by 13.

In the NFHS-4, information on the immunisation status for all children under 5 years of age in the household was collected through the interviewer’s review of the child’s immunisation card. However, in the absence of an immunisation card, information about immunisation status was gathered from the mother of the respective child on a recall basis. This is the standard procedure that the DHS adopts to collect information on immunisation coverage in other LMICs [11]. We based our coding for all four dependent variables on the immunisation card where available, and mother’s recall of immunisation only where the immunisation card was not available.

### Independent variables

We used several sociodemographic characteristics as key predictors of immunisation coverage. Children’s own characteristics may impact on caregivers’ health behaviour decision-making [12–14]. Based on information from mothers’ reports for each child, we considered the child’s sex (male, female), age of the child (in months), birth order (1-2, 2+), and type of birth (single, multiple).

Maternal characteristics may likewise impact on health knowledge and decision-making around children’s health [14–17]. Accordingly, we included unwanted pregnancy (no, yes), maternal education (no education, primary school, secondary school, higher secondary and above), and place of delivery (home delivery, institution delivery).

Finally, in light of previous research suggesting that household and community characteristics also impact on health decision-making, availability of resources for seeking preventive healthcare, and access barriers (e.g. distance to and quality of facilities), we also considered household factors. We included measures for place of residence (rural, urban), caste (scheduled caste/tribe, other backward class, others), religion (Hindu, Muslim, other), and household socioeconomic status (SES).

Typically, household SES is measured through income, consumption, or expenditure information. Adequate direct information on income and expenditures are not available in NFHS data, however. We therefore constructed an index of SES for each household using principal components analysis [18]. The following variables were used in our principal components analysis: having a radio, having a television, having a refrigerator, having a bicycle, having a motor-scooter, having a car, having a telephone, floor, wall and roof material, clean cooking, fuel having electricity, and mother’s education. From this analysis, household SES quintiles were calculated to create our measure of household SES.

### Analytical methods

#### Socioeconomic inequalities in child immunisation coverage

Similar to previous studies, we use the concentration index (CI) as our measure of socioeconomic inequality in immunisation coverage. In general, values of the CI can range from − 1 to + 1, with a value of zero indicating the absence of any socioeconomic inequality in the health outcome. A negative value indicates the disproportionate concentration of health indicator among the poor, while a positive value indicates the inverse. The CI is given by:

1 $$ CI=\frac{2}{\mu}\mathit{\operatorname{cov}}\left({y}_i{r}_i\right) $$

Where *y*_*i*_ is the health variable (in our study, calculated separately for each of our four dependent immunisation variables) of individual i and in SES quintile *r*_*i*_, divided by the mean immunisation (μ).

However, in the case of a binary outcome variable, the CI does not have the usual + 1/− 1 limits. In this study, the CIs were estimated as suggested by Wagstaff and Erreyger and Van Ourti for analysis of binary outcome variables. We have estimated age standardized CI. Standardization for age was required for our three binary indicators of immunisation as coverage of immunisation in our sample varies considerably across child age groups; immunisation intensity does not require normalisation [19, 20]. Full details of CI standardization are given in the supplementary file (S1).

#### Decomposing the concentration index of immunisation coverage

Although the CI shows the extent of socioeconomic-related inequalities in immunisation coverage, it cannot explain the factors that contributed to observed inequalities. To address this concern, we followed Wagstaff (2005) to decompose the CI in order to explain inequalities in immunisation coverage [21]. Details of this methodology are given in the supplementary file (S1). A probability of *p* ≤ 0.05 was considered significant; however, a Bonferroni correction for multiple comparisons was applied (0.05/12) in all of our models, so the effective *p*-value was *p* ≤ 0.0042. All analysis was completed using STATA v.14 software.

## Results

### Descriptive statistics

Table 2 show that there are 182,552 children included in the sample. 37% of children are fully immunised, 56% children are partially immunised, and 7% of children have never been immunised. No difference is found in the uptake of immunisation by gender. Average immunisation intensity is about 0.738 (see Supplementary Table S1 for the breakdown of immunisation intensity by sociodemographic characteristics).

**Table 2:** Percentage of coverage of immunisation status among children aged 12–59 months in India, 2015–16

	No immunisation	Partial immunisation	Full immunisation
**Sex of the child **			
Male	7.33	55.76	36.91
Female	7.64	55.16	37.20
**Age of child (in months)**			
12–23	5.58	50.74	43.68
24–35	6.88	52.73	40.39
36–47	8.09	57.66	34.26
48–59	9.34	60.66	30.00
**Birth order**			
1–2	6.07	53.27	40.65
2+	10.80	60.67	28.53
**Type of birth**			
Single	7.51	55.52	36.97
Multiple	5.34	52.01	42.65
**Unwanted pregnancy**			
No	7.51	55.42	37.07
Yes	6.62	57.01	36.37
**Place of delivery**			
Home	15.52	64.71	19.76
Institutional	5.34	53.00	41.69
**Education**			
No education	12.45	61.26	26.34
Primary	7.00	59.25	33.75
Secondary	5.11	52.13	42.75
Higher secondary and above	3.85	47.70	48.44
**Place of residence**			
Rural	7.99	56.78	35.23
Urban	6.17	52.13	41.71
**Caste**			
Scheduled caste/tribes	7.80	55.85	36.35
Other backward class	7.17	55.92	36.92
Others	7.67	53.86	38.48
**Religion**			
Hindu	6.72	55.65	37.63
Muslim	12.10	58.26	29.63
Others	5.88	42.75	51.37
**Wealth index**			
Poorest	11.90	61.40	26.71
Poor	8.08	58.64	33.28
Middle	6.07	54.91	39.02
Rich	5.09	51.88	43.03
Richest	3.95	46.04	50.01
**Region**			
North	7.30	47.81	44.90
Central	8.47	63.66	27.87
East	6.67	56.23	37.10
Northeast	13.73	62.63	23.64
West	10.71	55.99	33.30
South	3.82	44.89	51.29
**Total**	**7.48**	**55.47**	**37.05**

**Fig. 1:**
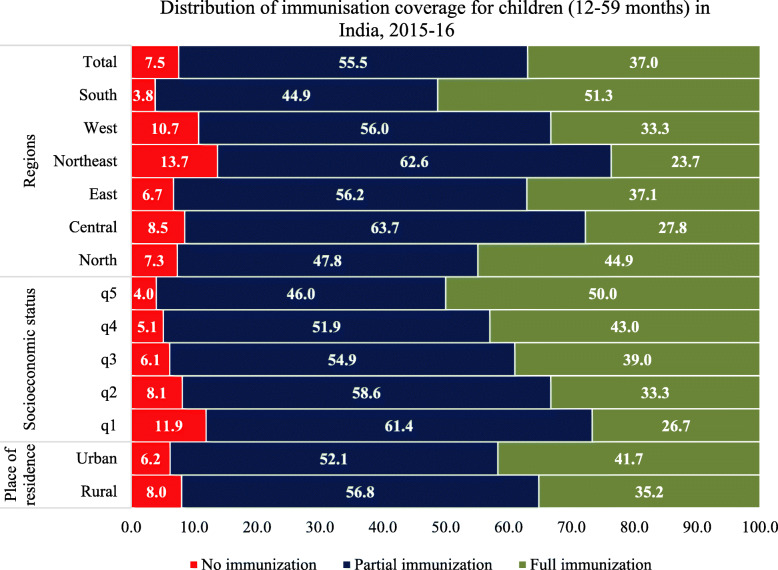
Distribution of **immunisation** coverage for children (12–59 months)

Figure 1 shows the distribution of immunisation coverage among children (aged 12–59 months) by selected sociodemographic characteristics. There is considerable variation in childhood immunisation across all regions and socioeconomic groups of India. The southern region has the highest proportion of fully immunised children (51.3%) and the lowest number of non-immunised (38%) children, while children in the north-eastern, western and central region of India have the lowest proportion of fully immunised children (23.7, 27.8 and 33.3% respectively) but the highest proportion of partially immunised children (62.6, 63.7 and 56.0% respectively). Figure 1 also depicts a socioeconomic gradient in immunisation coverage, with higher proportions of fully immunised children in households in the highest wealth quintiles, while a higher proportion of never immunised children are in lower wealth quintile households (11.9%). The coverage of fully immunised children is also higher among children from urban (41.7%) compared to rural settings (35.2%), a statistically significant difference (Pearson χ2 (1) = 642.7756, Pr = 0.000).

### Concentration index

Unstandardized and age standardized CI results for our three binary indicators for fully immunised, partially immunised, and never immunised children are provided in Tables 3, 4 and 5 respectively. (Results for immunisation intensity are not presented in this section because normalization is only required for binary indicators, as noted in the Methods section above.) Table 3 shows that the unstandardized and age standardized CI for fully immunised children is 0.136 and 0.131 respectively, which means that children belonging to highest wealth quintile (q5) are more likely to be immunised compared to children in the lowest wealth quintile (q1). This relationship is statistically significant for both the unstandardized and age standardized CIs. Furthermore, the indirectly age standardized CI based on Wagstaff normalization (Wc) and Erreyger’s normalization (Ec) also gives the significant estimate as 0.202 and 0.185 respectively. The CI confirms the pattern of pro-rich inequality across all the regions and by place of residence suggested by the descriptive statistics.

**Table 3:** CI’s for full immunisation of children (12–59 months) in India, 2015–16

	Unstandardized CI (SE)	Indirectly Age standardized CI(SE)	Wc Normalized (SE)	Ec Normalized (SE)
**Region**				
North	0.162*(0.003)	0.142*(0.003)	0.220*(0.005)	0.201*(0.004)
Central	0.153*(0.003)	0.144*(0.004)	0.223*(0.006)	0.204*(0.006)
East	0.087*(0.003)	0.071*(0.004)	0.110*(0.006)	0.100*(0.006)
Northeast	0.160*(0.006)	0.147*(0.007)	0.227*(0.011)	0.208*(0.010)
West	0.098*(0.007)	0.082*(0.007)	0.127*(0.011)	0.116*(0.010)
South	0.048*(0.004)	0.038*(0.004)	0.060*(0.006)	0.055*(0.006)
**Place of Residence**				
Rural	0.137*(0.002)	0.120*(0.002)	0.186*(0.003)	0.170*(0.003)
Urban	0.111*(0.003)	0.084*(0.003)	0.131*(0.005)	0.120*(0.004)
**Overall**	0.136*(0.003)	0.131*(0.002)	0.202*(0.003)	0.185*(0.003)

Note: *Significant at 5% (a Bonferroni correction has been applied); **SE**-standard error; **Wc-**indirectly standardized CI based on Wagstaff’s normalization**; Ec-** indirectly standardized CI based on Erreyger’s normalization

**Table 4:** CI’s for partial immunisation of children (12–59 months) in India, 2015–16

	Unstandardized CI (SE)	Indirectly standardized CI		
		Age standardized CI (SE)	Wc Normalized (SE)	Ec Normalized (SE)
**Region**				
North	−0.101*(0.003)	− 0.088*(0.003)	− 0.201*(0.007)	− 0.198*(0.007)
Central	− 0.038*(0.002)	−0.038*(0.001)	− 0.087*(0.002)	− 0.086*(0.002)
East	− 0.033*(0.003)	− 0.028*(0.003)	− 0.063*(0.007)	− 0.062*(0.007)
Northeast	− 0.008*(0.003)	− 0.010*(0.003)	− 0.024*(0.007)	− 0.023*(0.007)
West	− 0.033*(0.004)	− 0.028*(0.004)	− 0.063*(0.009)	−0.062*(0.009)
South	−0.047*(0.005)	−0.038*(0.005)	− 0.087*(0.011)	−0.085*(0.011)
**Place of Residence**				
Rural	−0.055*(0.001)	−0.051*(0.001)	− 0.116*(0.002)	−0.114*(0.002)
Urban	−0.064*(0.003)	−0.050*(0.002)	− 0.115*(0.005)	−0.113*(0.004)
**Overall**	−0.062*(0.002)	−0.061*(0.001)	− 0.140*(0.002)	−0.138*(0.002)

Note: *Significant at 5% (a Bonferroni correction has been applied); **SE**-standard error; **Wc-**indirectly standardized CI based on Wagstaff’s normalization**; Ec-** indirectly standardized CI based on Erreyger’s normalization

**Table 5:** CI’s for never immunisation of children (12–59 months) in India, 2015–16

	Unstandardized CI	Indirectly standardized CI		
		Age standardized CI	Wc Normalized	Ec Normalized
**Region**				
North	−0.356*(0.011)	−0.335*(0.012)	− 0.366*(0.013)	− 0.113*(0.004)
Central	− 0.214*(0.008)	− 0.206*(0.008)	− 0.225*(0.009)	− 0.070*(0.003)
East	− 0.198*(0.011)	− 0.169*(0.011)	− 0.184*(0.012)	− 0.057*(0.004)
Northeast	− 0.212*(0.009)	− 0.193*(0.008)	− 0.211*(0.009)	− 0.065*(0.003)
West	− 0.130*(0.014)	−0.117*(0.015)	− 0.128*(0.016)	− 0.040*(0.005)
South	−0.088*(0.022)	−0.075*(0.021)	− 0.082*(0.023)	−0.025*(0.007)
**Place of Residence**				
Rural	−0.203*(0.005)	−0.179*(0.004)	− 0.196*(0.004)	−0.061*(0.001)
Urban	−0.208*(0.011)	−0.161*(0.010)	− 0.176*(0.011)	−0.054*(0.003)
**Overall**	−0.207*(0.007)	−0.206*(0.004)	− 0.225*(0.004)	−0.070*(0.001)

Note: *Significant at 5% (a Bonferroni correction has been applied); **SE**-standard error; **Wc-**indirectly standardized CI based on Wagstaff’s normalization**; Ec-**indirectly standardized CI based on Erreyger’s normalization

Table 4 shows the CI results for partial immunisation, which is different from full immunisation, where CIs are consistently negative and statistically significant at the 95% confidence interval for both unstandardized and age standardized CI. The results from the Wagstaff normalization (− 0.140) and Erreyger’s normalization (− 0.138) also confirm that partial immunisation is more concentrated among poorer than richer children. A similar pattern is observed by place and region of residence for partially immunised children.

Table 5 shows the unstandardized (− 0.207) and age standardized (− 0.206) CIs for never immunised children. Never immunised children are disproportionately in poorer households, and this relationship is statistically significant at the 95% level. Wagstaff (− 0.225) and Erreyger’s normalization (− 0.070) processes confirm this socioeconomic gradient in immunisation. Of the over one-third (37%) of children in India who are never immunised, a higher proportion live in rural (− 0.179) compared to urban areas (− 0.161). Wagstaff normalization shows non-immunisation is also concentrated among poorer households across all regions. The North, Central and Northeast regions in particular have a significant concentration of never immunised children among the poor (Table 5). A relatively smaller but similar pattern of concentration has been found across all regions for non-immunisation using Erreyger’s normalization process.

### Decomposition of socioeconomic inequality

Table 6 presents the decomposition analysis based on the ordinary least square (OLS) regression, which indicates the elasticity, CI, and contribution of each covariate to overall inequality for full, partial, and never immunisation, and for immunisation intensity. Each of these outcomes is modelled separately in the results presented in Table 6. Contributors to disparities in immunisation coverage based on the results of the decomposition analysis include age, gender, birth order, multiple births, pregnancy intention, place of delivery, place of residence, mother’s literacy, caste, religion, household wealth, and region.

**Table 6:** Decomposition of CI for immunisation status (full /partial/never) among children below age 5 years in India

	Model 1 Full Immunisation	Model 2 Partial Immunisation		Model 3 Never Immunisation		Model 4 Immunisation intensity		
	Elasticity (C.I.)	Contribution (%)	Elasticity (C.I.)	Contribution (%)	Elasticity (C.I.)	Contribution (%)	Elasticity (C.I.)	Contribution (%)
Female Child	0.007(− 0.011)	0.000(− 0.001)	− 0.006*(− 0.011)	0.000(− 0.001)	0.010(− 0.011)	0.000(0.001)	− 0.001(− 0.011)	0.000(0.000)
Age of child	− 0.160*(0.002)	0.000(0.003)	0.078*(0.002)	0.000(0.004)	0.205*(0.002)	0.000(0.002)	−0.029*(0.004)	0.000(0.002)
Birth order > = 2	−0.033*(− 0.223)	0.007(0.053)	0.016*(− 0.223)	−0.003(0.056)	0.045*(− 0.223)	−0.010(0.048)	− 0.011*(− 0.225)	0.002(0.061)
Multiple birth	0.002*(0.075)	0.000(0.001)	−0.001(0.075)	0.000(0.001)	−0.003*(0.075)	0.000(0.001)	0.000*(0.075)	0.000(0.001)
Unwanted pregnancy	−0.002(− 0.006)	0.000(0.000)	0.002*(− 0.006)	0.000(0.000)	− 0.002*(− 0.006)	0.000(0.000)	0.000*(− 0.005)	0.000(0.000)
Institutional delivery	0.310*(0.093)	0.029(0.213)	− 0.100*(0.093)	− 0.009(0.151)	− 0.780*(0.093)	− 0.073(0.349)	0.115*(0.092)	0.011(0.269)
Education	0.109*(0.565)	0.023(0.169)	−0.029*(0.565)	− 0.009(0.139)	− 0.309*(0.565)	− 0.046(0.222)	0.057*(0.558)	0.010(0.262)
Urban residence	−0.0140*(0.445)	− 0.006(− 0.045)	0.005(0.445)	0.002(− 0.034)	0.030*(0.445)	0.013(− 0.064)	− 0.005*(0.443)	− 0.002(− 0.062)
**Caste**								
Other backward caste	0.002(− 0.320)	0.000(− 0.002)	− 0.004*(− 0.324)	0.001(− 0.019)	0.021*(− 0.324)	−0.007(0.033)	− 0.002*(− 0.325)	0.001(0.017)
Others	− 0.013*(0.219)	− 0.003(− 0.002)	0.001*(0.023)	0.000(− 0.001)	− 0.020*(0.023)	0.000(0.002)	− 0.002*(0.024)	0.000(− 0.002)
**Religion**								
Muslim	− 0.020*(− 0.015)	−0.001(− 0.004)	0.002(− 0.012)	0.000(0.000)	0.082*(− 0.012)	− 0.001(0.005)	− 0.010*(− 0.014)	0.000(0.004)
Others	0.003(0.021)	0.000(0.000)	−0.008*(0.253)	− 0.002(0.032)	0.002*(0.253)	0.001(− 0.002)	0.001(0.254)	0.000(0.008)
**Wealth index**								
Poor	0.013*(− 0.246)	−0.003(− 0.023)	−0.002(− 0.246)	0.001(− 0.009)	−0.046*(− 0.245)	0.011(− 0.054)	0.006*(− 0.249)	−0.002(− 0.038)
Middle	0.027*(0.137)	0.004(0.028)	−0.010*(0.140)	− 0.001(0.023)	− 0.059*(0.14)	−0.008(0.04)	0.009*(0.136)	0.001(0.032)
Rich	0.038*(0.465)	0.018(0.133)	−0.016*(0.468)	−0.008(0.126)	− 0.066*(0.468)	−0.031(0.148)	0.011*(0.465)	0.005(0.128)
Richest	0.053*(0.743)	0.039(0.294)	−0.026*(0.746)	−0.019(0.317)	− 0.067*(0.746)	−0.050(0.239)	0.013*(0.742)	0.009(0.239)
**Residual**		**0.021(0.166)**		**−0.013(0.212)**		**−0.006(0.031)**		**0.003(0.081)**
**Total**		**0.134**		**−0.061**		**−0.208**		**0.039**

Note: 1) * Significant at 5% (a Bonferroni correction has been applied) **2)** All models include region fixed effect

For all four immunisation outcomes, the largest contributions to inequalities in immunisation status come from household wealth, followed by institutional delivery and mother’s education. Household wealth contributed to 48% of the inequality in full immunisation and 42% of the inequality in never immunisation, while institutional delivery contributed to 21% of the inequality in full immunisation and 36% of the inequality in never immunisation. Mother’s education contributed to 14% of the inequality in full immunisation and 25% of the inequality in never immunisation. Household wealth, institutional delivery, and maternal education contribute about 51, 14 and 9% of the inequality in partial immunisation respectively. Overall, the socioeconomic determinants included in our model explain 89 and 84% of the inequality in full and partial immunisation respectively. The same socioeconomic determinants explain about 100% inequality in never immunisation and immunisation intensity. By inference, this means that the residual, or inequality in full immunisation explained by other factors, is 1.5%.

The decomposition analysis also reveals the that crucial factors that are responsible for an increase in the concentration of fully immunised children among wealthier households are institutional delivery, mother’s literacy, urban residence and geopolitical location. Similar factors are observed to be important for the concentration of partially and never immunised children among poorer households. These results identify mother’s education, household wealth, and place of delivery as imperative variables in explaining inequalities in children’s immunisation.

## Discussion

Using nationally representative data from the latest round of the NFHS (2015–16), we examined the extent of inequalities in childhood immunisation (12–59 months) in six regions and five socioeconomic groups in India, with focus on factors associated with full, partial, and no immunisation in India. Overall, we found strong evidence of a socioeconomic and geographic gradient in immunisation, with fully immunised children disproportionately concentrated in households in the highest wealth quintiles, and residing in urban areas and the southern and western regions of the country. Meanwhile, we found that partially and never immunised children are in lower wealth quintiles, rural areas, and northern regions. These findings corroborate those of previous studies from other countries, and earlier studies from India [8, 22, 23]. We also examined socioeconomic inequality in immunisation intensity in our analysis. Inclusion of immunisation intensity is important as it uncovers the factors associated with the additive receipt of more vaccines among partially immunised children. Findings indicate a concentration of higher immunisation intensity among the highest wealth quintile. This complements a similar finding reported in a previous study from Nigeria [24].

Our findings suggest that mother’s education is an important factor that contributes to the disparities in immunisation coverage across different socioeconomic groups, different regions, and by place of residence. This finding is consistent with the earlier studies from India [25–28] that argue that higher maternal education leads to an increase in the utilisation of health care services, which in turn facilitates vaccine uptake. Our complementary findings suggest that the inequalities in immunisation found here are attributable to changes in level of maternal education, place of residence, geographical region, and wealth quintile. Nor are these factors independent of one another: the proportion of illiterate mothers is higher in rural (36%) than urban (16%) areas, and poorer households have a higher proportion of illiterate mothers (63%), while richer households have a lower proportion of illiterate mothers (4%). Geographic regions with poor immunisation coverage (western, north-eastern and some parts of the west and central regions) also have a higher proportion of illiterate mothers.

Our study shows that full vaccination coverage is more prevalent among well-off households, while partial and non-immunisation are more prevalent among economically deprived households. This finding is consistent with previous studies which also show that higher socioeconomic status is associated with higher immunisation rates [29].

We also identified place of residence as an important factor that contributes to the disparities in childhood immunisation in India. Research using previous rounds of the NFHS has also found low coverage of immunisation among children in rural compared to urban locations [10, 30–32]. It has been suggested that, in rural areas, rates of full immunisation can be improved by increasing health services through immunisation camps and providing incentives to mothers [33]. These findings highlight expanded health facilities and broader improvements to the health system as an important supply-side function. Previous research also suggests there may be some demand-side functions (acceptability factors, such as culture and expectations that parents have of the health system) which could also affect the uptake of maternal and child healthcare in general [34, 35] and immunisation in particular [36, 37].

Our findings also highlight considerable regional disparities in non-immunisation across the country. In general, the states with the lowest proportion of fully immunised children are clustered in the central, eastern, north-eastern (except Sikkim) regions, and a few states of the western region. On the other end of the spectrum, all of the southern states are performing very well on immunisation coverage, with high rates across the region. An encouraging finding from our study is the improvement in functionality of healthcare system with respect to immunisation, reflected by the relatively small percent of non-immunised children compared to previous periods.

One further factor strongly associated with the wide disparity in immunisation coverage in our study is the use of institutional delivery facilities for childbirth. There are several possible explanations of this association, which are not mutually exclusive: First, Janani Suraksha Yojana (JSY), India’s conditional cash transfer program which aims to reduce the maternal mortality ratio through promotion (using cash incentives) of institutional deliveries, has improved reproductive and child health-related indicators in India [38]. Although childhood vaccines are scheduled up to the age of 12 months, several vaccines, such as polio, hepatitis B, and BCG, take place at the time of birth [39]; interaction with community health workers through JSY and institutional delivery may facilitate vaccination at birth. Second, women’s interaction with health care providers and the health system may increase the awareness of other health issues which can occur in the post-partum period, and thereby encourage continued care throughout the child’s first years of life.

There are many unexplained factors associated with partial vaccination and non-vaccination among Indian children which we were not able to explore in this study. For instance, one study in northern India found that a substantial proportion of mothers (12%) failed to give a specific reason for why their children had not received vaccinations [40]. Dropout rates of some or all vaccines raise several questions about supply and demand-side factors. One study examined the reason behind partial and non-immunisation of children in Lucknow district in India. Lack of knowledge and lack of faith were found to be the main reasons for non-immunisation of children [41]. Additional demand-side factors include a knowledge gap about the benefits of immunisation, mothers’ limited awareness of childhood immunisation schedules and sources, and lack of exposure to the media [42, 43].

Regional disparities in non-immunisation and partial immunisation can be attributed not only to demand, but also to supply-side factors. India’s vaccine deficit system may therefore also be a reason for non-vaccination or partial vaccination of children [44]. Supply-side factors include failure of health workers to arrive on time and/or reliably and inadequate supplies of vaccines [45]. Even though immunisation is free in India, travel costs and opportunity costs associated with waiting times can be high, and may be seen as particularly insurmountable for female children and families residing in rural areas [11].

### Strengths and limitations

This study has several key strengths. First, we used a large sample from the most recent round of the NFHS-4 (2015–16) to examine the socioeconomic inequalities in coverage of child immunisation and factors associated with these inequalities. This study therefore provides up-to-date, population level information on childhood immunisation coverage in India. Second, for the first time, we also examined the inequalities in non-immunisation, and assessed the factors associated with inequalities in non-immunisation. This is important because previous research suggests that factors driving non-immunisation may differ substantively from those driving partial immunisation [42].

As with all observational studies, this study also has some limitations. One limitation is that vaccination variables were estimated by combining data from immunisation cards and information from the mothers’ recall when immunisation cards were not available. Mothers’ reporting on child immunisation may subject to both recall and social desirability bias. In our study, an immunisation card was available for about half of the sample, with information regarding immunisation for the remaining 50% being based on mother’s recall. Moreover, unavailability of vaccination cards was also higher among children from households from the lowest wealth quintile, with less educated mothers, and among those residing in rural areas. Another limitation of the study is that we were unable to measure health systems directly. While SES, urban residence, and region are correlated with differences in health systems, there may be other structural factors we are not able to capture with the data.

## Conclusions

Findings from this study provide evidence of continuing, current socioeconomic inequalities in child immunisation in India, and document factors associated with these inequalities. From a policy and health systems planning perspective, our study is useful to address the challenge of low immunisation and to eliminate vaccine-preventable deaths.

## Supplementary information

**Additional file 1: Table S1.** Average immunisation intensity among children aged 12–59 months by background characteristics in India, 2015–16.

## Data Availability

The data can be downloaded from the website of the Demographic and Health Survey (DHS) (https://dhsprogram.com/data/). The data for the current study were downloaded from the aforementioned website after receiving permission.

## Abbreviations

NFHS: National Family Health Survey
WHO: World Health Organization
UNICEF: United Nations Children’s Fund
MDGs: Millennium Development Goals
SES: Socioeconomic Status
