# Supplementary material for: Explaining socioeconomic inequalities in immunisation coverage in India: new insights from the fourth National Family Health Survey (2015–16)
Source: BMC Pediatr. 2020 Jun 16;20:295. doi: 10.1186/s12887-020-02196-5 (PMC7296926; doi:10.1186/s12887-020-02196-5)
Supplement: Supplementary file 1 — Additional file 1: Table S1. Average immunisation intensity among children aged 12–59 months by background characteristics in India, 2015–16. [file 12887_2020_2196_MOESM1_ESM.docx]

***Supplementary file (S1)***

***Analytical methods***

***Socioeconomic inequalities in child immunisation coverage***

Following Atanguba et al. ([Ataguba et al., 2016](#_ENREF_1)), we used the concentration index (CI) to measure the inequality in childhood immunisation coverage in India. The CI is widely used to measure socioeconomic inequalities in health outcomes. Values can range from -1 to +1, with a value of zero indicating the absence of any socioeconomic inequality in the health outcome. A negative value indicates the disproportionate concentration of the health indicator (here immunisation coverage) among the poor, while a positive value indicates the inverse. The CI is given by:

$$CI=\frac{2}{\mu}cov\left( y_{i},r_{i} \right) \ldots\ldots\ldots\ldots(1)$$

Where $y_{i}$ is the health variable (all mutually exclusive category of immunisation) of individual i and ranking of socio-economic status $r_{i}$, divided by the mean immunisation (µ).

We also estimated standardized CIs. Standardization accounts for the possibility that specific sociodemographic variables such as sex or age are correlated with either the health measure of interest, SES, or both. There are two possible methods of standardization: **1) direct standardization**, which provides the distribution of immunisation coverage across SES groups that would be observed if all groups had the same sociodemographic structure; and **2) indirect standardization**, which ‘corrects’ the actual distribution of immunisation coverage by comparing it with the distribution that would be observed if all individuals had their own sociodemographic distribution of children (15).

In this study, we used the indirect method of standardization for each immunisation category (full immunisation, partial immunisation and non-immunisation) according to age. The most natural way to indirectly standardized a health variable through simple regression is given by:

$y_{i}=\alpha+\sum_{j} \beta_{j}x_{ji}+\sum_{k} \gamma_{k}z_{ki}+\varepsilon_{i}$ $\ldots\ldots\ldots(2)$

where $y_{i}$ is some indicator of health (in our case categories of full immunisation/ partial immunisation/ non-immunisation); *i* denotes the individual; and α*,* β*,* and γ are parameter vectors. The $x_{j}$ are confounding variables which the study seeks to standardize (in our case age of the child), and the $z_{k}$ are non-confounding variables for which study do *not* want to standardize but to control for to estimate partial correlations with the confounding variables. If the study seeks to standardize for the full correlations with the confounding variables, the $z_{k}$ variables are left out of the regression. Ordinary least squares (OLS) parameter estimates ( $\hat{\alpha},\hat{\beta}_{j},\hat{\gamma}_{k} )$ individual values of the confounding variables ($x_{ji}$), and sample means of the non-confounding variables ( $\bar{z}_{k}$) are then used to obtain the predicted values of the health indicator $\hat{y}_{i}^{X}-$

$$\hat{y}_{i}^{X}=\hat{\alpha}+\sum_{j} \hat{\beta}_{j}x_{ji}+\sum_{k} \hat{\gamma}_{k}\bar{z}_{k}+\varepsilon_{i} \ldots\ldots\ldots(3)$$

Estimates of an indirectly standardized outcome variable are given by-

$$\hat{y}_{i}^{IS}=\hat{y_{i}}-\hat{y}_{i}^{X}+\overline{y}\ldots\ldots\ldots(4)$$

As mentioned above, the value of CI usually lies between -1 to +1, but in this case, where the outcome variables are binary (0,1), the the bounds of CI are not -1 and +1 but depend on the mean of the variable. Recently, there has been a debate regarding the appropriate normalization process between Wagstaff and Erreygers about the matter of bounds of the CI ([Wagstaff, 2005](#_ENREF_3), [Wagstaff, 2009](#_ENREF_4)).

According to Wagstaff, in the case of binary variable, the CI does not have the usual limits. It should lie between (μ-1) and (1- μ) and will require the normalization by (1- μ). Here μ is the mean of the outcome variable ([Wagstaff, 2005](#_ENREF_3)). However, Erreygers proposed another way to adjust the CI in the case of a binary outcome variable ([Erreygers, 2009](#_ENREF_2)). Erreygers (2009) adjusted the CI $E_{c}$ which is equivalent to

$$E_{c}=4\left( \frac{\mu}{b-a} \right).CI\ldots\ldots\ldots.\ldots\ldots\ldots(5)$$

Where (b-a) is the range of the variable of interest.

Wagstaff’s (2005) normalized index $W_{c}$ is given as

$$W_{c}=\frac{CI}{1-\mu} \ldots\ldots\ldots.\ldots\ldots\ldots(6)$$

Ataguba et al. in 2011 shows that in case of binary variable the Erreygers index $E_{c}$ can be written equivalently as (Ataguba et al., 2011)-

$$E_{c}=4W_{c}\left( \mu-\mu^{2} \right)\ldots\ldots\ldots.\ldots\ldots\ldots(7)$$

Following from this debate, our study standardized the CI in two ways. First, adjusting only for age, and second, adjust for age and another confounding variable. We did not find any difference between the age-standardized CI and age confounding standardized CI, therefore, selected the latter approach.

**Decomposing the CI of immunisation coverage**

Although the CI shows the extent of socioeconomic-related inequalities in immunisation coverage, it cannot explain the factors that contributed to observed inequalities. To address this concern, we followed Wagstaff et al. (2005) to decompose the CI in order to explain inequalities in immunisation coverage ([Wagstaff et al., 2003](#_ENREF_5)). For any linear additive regression model of outcome variable (*y*), such as

$y_{i}=\alpha+\sum_{k} \beta_{k}x_{k}+\varepsilon_{i} \ldots\ldots\ldots(8)$

The CI for the outcome variable y can be written as

$$CI=\sum_{k} \left( \frac{\beta_{k}\bar{x}_{k}}{\mu} \right).{CI}_{k}+\frac{GC_{\varepsilon}}{\mu} \ldots\ldots\ldots(9)$$

Where $\mu$ is the mean of the health variable (full/partial/never immunisation in our study); the index *k* refers to the regressors included in the equation; $\beta_{k}$ is the coefficient for each of the health determinants from equation 10; $\bar{x}_{k}$ is the mean of each of the regressors; and $GC_{\varepsilon}$ is the generalized CI for the error term $\varepsilon_{i}$ (Wagstaff et al., 2007).

Equation (9) shows that *CI* can be thought of as being made up of two components: the deterministic component $(\sum_{k} \left( \frac{\beta_{k}\bar{x}_{k}}{\mu} \right).{CI}_{k}$ ) and a residual component ( $\frac{GC_{\varepsilon}}{\mu}$ ). The contribution of each$x_{k}$ (explanatory variable) to explained inequality (inequality in immunisation status) is derived by multiplying the elasticity component by the corresponding CI ${CI}_{k}$. Therefore, if the coefficient estimates for $\beta_{k}$ are not statistically different from zero, then the contribution of the particular variable to explain inequalities will also not be statistically significant. The residual component reflects the inequality that cannot be explained by systematic variation across economic development in the determinants of the $x_{k}$variables.

***Supplemental Results***

Below, we present the average immunisation intensity by background characteristics for our sample.

| **Table S1**: Average immunisation intensity among children aged 12-59 months by background characteristics in India, 2015-16. | |
| --- | --- |
|  | Immunization intensity |
| **Gender** |  |
| Male | 0.739 |
| Female | 0.737 |
| **Age of child (in months)** |  |
| 12-23 | 0.735 |
| 24-35 | 0.744 |
| 36-47 | 0.740 |
| 48-59 | 0.734 |
| **Birth order** |  |
| 1-2 | 0.752 |
| 2+ | 0.712 |
| **Type of birth** |  |
| Single | 0.738 |
| Multiple | 0.741 |
| **Pregnancy intention** |  |
| Wanted | 0.740 |
| Unwanted | 0.709 |
| **Place of delivery** |  |
| Home | 0.693 |
| Institutional | 0.756 |
| **Education** |  |
| No | 0.700 |
| Primary | 0.729 |
| Secondary | 0.761 |
| Higher | 0.791 |
| **Place of residence** |  |
| Rural | 0.734 |
| Urban | 0.750 |
| **Caste** |  |
| Scheduled caste/tribes | 0.731 |
| Other backward caste | 0.738 |
| Others | 0.756 |
| **Religion** |  |
| Hindu | 0.747 |
| Muslim | 0.709 |
| Others | 0.720 |
| **Wealth index** |  |
| Poorest | 0.708 |
| poor | 0.727 |
| Middle | 0.750 |
| Richer | 0.762 |
| Richest | 0.781 |
| **Region** |  |
| North | 0.733 |
| Central | 0.716 |
| East | 0.775 |
| Northeast | 0.719 |
| West | 0.748 |
| South | 0.777 |
| **Total** | **0.738** |

**References**

Ataguba JE, Ojo KO & Ichoku HE 2016. Explaining socio-economic inequalities in immunization coverage in Nigeria. *Health policy and planning,* 31**,** 1212-1224.

Erreygers G 2009. Correcting the concentration index. *J Health Econ,* 28**,** 504-15.

Wagstaff A 2005. The bounds of the concentration index when the variable of interest is binary, with an application to immunization inequality. *Health economics,* 14**,** 429-432.

Wagstaff A 2009. Correcting the concentration index: a comment. *Journal of Health Economics,* 28**,** 516-520.

Wagstaff A, Van Doorslaer E & Watanabe N 2003. On decomposing the causes of health sector inequalities with an application to malnutrition inequalities in Vietnam. *Journal of econometrics,* 112**,** 207-223.
